# Chronic Tonsillitis as a Focal Infection: A Decade-Long Case Involving Severe Systemic Symptoms

**DOI:** 10.7759/cureus.100929

**Published:** 2026-01-06

**Authors:** András Lakos

**Affiliations:** 1 Outpatient Service, Center for Tick-borne Diseases, Budapest, HUN

**Keywords:** arthralgia, chronic tonsillitis, false positive borrelia test, focal disease, focal infection, sore throat, tonsillar detritus, tonsillar stone, tonsillectomy

## Abstract

Unlike acute tonsillitis, which is readily recognized as infectious, chronic tonsillitis, tonsilloliths, and tonsillar detritus are often considered non-infectious and benign, despite their potential to act as focal infections causing systemic inflammatory symptoms that are frequently overlooked when tonsillar compression is not performed. We report the case of a 29-year-old female with a decade-long history of progressive musculoskeletal pain, episodic low-grade fever, headaches, and exercise-induced inflammatory arthralgia affecting the feet, ankles, wrists, and spine.

The initial otolaryngologic (ENT) evaluation revealed purulent material expressed on tonsillar compression, and tonsillectomy was recommended but deferred. Over subsequent years, the patient developed chronic plantar fasciitis, seronegative polyarthritis, and widespread pain, leading to multiple rheumatologic and autoimmune diagnoses, including an undifferentiated autoimmune syndrome. Repeated ENT examinations were largely unremarkable because tonsillar compression was not performed, and tonsillar stones or detritus were repeatedly dismissed as clinically insignificant. Laboratory investigations showed no sustained systemic inflammation, and repeated Borrelia serology yielded false-positive IgM results, prompting referral for suspected Lyme disease. Focused re-evaluation identified chronic tonsillitis as a focal infectious source. Tonsillectomy was performed a decade after symptom onset. Following surgery, the patient experienced a gradual and complete resolution of all symptoms and has remained symptom-free on long-term follow-up through the end of 2025, despite prior skepticism regarding the potential benefit of the procedure.

This report demonstrates that chronic tonsillitis, including tonsillar stones and detritus, can act as a focal infection capable of causing severe and persistent systemic inflammatory symptoms even in the absence of overt local signs or laboratory abnormalities. Failure to distinguish chronic tonsillitis from recurrent acute bacterial tonsillitis may result in prolonged morbidity and diagnostic error. Manual tonsillar compression is essential for accurate diagnosis, and tonsillectomy can be curative even after years of symptoms. Greater clinical awareness of oral and tonsillar focal infections is needed to prevent unnecessary diagnostic delays and inappropriate treatment.

## Introduction

Chronic tonsillitis is a persistent inflammation of the tonsils that differs from recurrent acute throat infections, which are typically short-lived episodes of bacterial infection. Unlike acute infections, chronic tonsillitis may cause long-term, often nonspecific symptoms that involve multiple body systems. Importantly, these systemic manifestations can be overlooked even when patients consult an ENT specialist, potentially leading to delayed diagnosis and inappropriate management. Because patients presenting with heel pain, spinal pain, or other nonspecific systemic symptoms rarely consult an otorhinolaryngologist, the systemic consequences of tonsillar focal infections often remain unrecognized. Tonsillar detritus, although commonly regarded as a benign finding, contains significant bacterial material [1]. Tonsillar stones are generally considered to result from recurrent episodes of acute tonsillitis rather than an ongoing chronic inflammatory process [2,3]. Similarly, tonsilloliths are widely viewed as harmless; however, microbiological analyses indicate that they represent biofilm-based calcified abscesses [4].

Calcification in this context may reflect a host defensive response to persistent infection, analogous to calcium deposition observed in other chronic infectious diseases, including filariasis [5], cysticercosis [6], neurocysticercosis [7], tuberculosis [8], and toxoplasmosis [9]. Despite these parallels, chronic tonsillitis as a distinct clinical entity remains poorly characterized and is frequently underestimated in routine practice. A notable illustration of this uncertainty is found in a recent review that describes chronic tonsillitis as an “obsolete” term and recommends tonsillectomy solely based on the frequency of acute inflammatory episodes [10]. In clinical practice, chronic tonsillitis may be overlooked because standard ENT examinations do not always include manual compression of the tonsils, a procedure that is often necessary to express purulent material from subepithelial crypts. As a result, the fundamental clinical differences between recurrent acute tonsillitis and chronic tonsillitis may remain unclear (see Appendices).

Observations from a specialized outpatient service dedicated to tick-borne diseases, with clinical experience spanning several decades, indicate that many patients evaluated for suspected chronic Lyme disease instead suffer from focal infections. These patients frequently report similar, largely subjective symptoms, which increases the risk of diagnostic misattribution and may lead to treatment with sedatives or referral for psychiatric evaluation. Within this clinical setting, symptom resolution has often been observed following tonsillectomy, including in cases with long-standing complaints. While the inclusion of epidemiological data would enhance the introduction, reliable prevalence estimates are not available due to the lack of a clear distinction between chronic tonsillitis and recurrent acute bacterial tonsillitis in the literature. Nevertheless, highlighting the potential systemic impact of chronic tonsillitis provides valuable clinical context and educational value.

## Case presentation

The patient, a Hungarian woman who later relocated to Austria, was 29 years old when her symptoms first became clinically significant. Her medical history included successfully treated endometriosis, with no other notable conditions. In 2011, because of allergic rhinitis and recurrent sore throats, she consulted a Hungarian otolaryngologist. Purulent discharge was observed upon tonsillar compression, and tonsillectomy was recommended; however, the risks of not undergoing surgery were not communicated, and the procedure was postponed. The patient’s illness followed a slow, progressive course, characterized by fluctuating but increasingly disabling symptoms, progressing from localized complaints to a multisystem inflammatory syndrome. Over several years, symptom severity and functional impairment increased despite repeated specialist consultations, while routine investigations repeatedly failed to reveal a unifying cause.

The patient's daily life became progressively limited by unpredictable inflammatory pain affecting the ankles and wrists, often triggered by minimal physical exertion. Periods of musculoskeletal pain were accompanied by severe headaches brought on by ordinary movements such as bending forward, as well as episodes of palpitations and shortness of breath that caused persistent anxiety despite normal cardiologic findings. At times, spinal pain and joint involvement were so debilitating that the patient required crutches to walk and was intermittently confined to bed. These symptoms substantially impaired mobility, independence, and overall quality of life, while the absence of a clear diagnosis contributed to emotional distress and uncertainty regarding prognosis and treatment.

Clinical attention focused primarily on excluding rheumatologic, neurologic, and cardiovascular disease, leading to multiple provisional diagnoses but no sustained therapeutic benefit. Otolaryngologic assessments largely concentrated on the absence of overt local inflammation, which contributed to the tonsils being excluded as a potential etiologic source. Management strategies were therefore predominantly conservative or symptomatic, influenced partly by life circumstances that limited acceptance of proposed immunomodulatory therapies. This prolonged period of diagnostic uncertainty and therapeutic fragmentation ultimately delayed recognition of a focal infectious origin, despite the persistence and progression of systemic symptoms. The complete timeline of symptoms, diagnostic activity, and treatment strategy is presented in Table 1.

**Table 1:** Timeline of symptoms, diagnostic activity, and treatment strategy MRI: magnetic resonance imaging; SLE: systemic lupus erythematosus

Year/period	Clinical events and symptoms	Evaluations and diagnoses	Treatment strategy and outcome
2011	Recurrent sore throats and allergic rhinitis	ENT evaluation in Hungary; purulent material expressed from tonsils	Tonsillectomy recommended but postponed; no etiological treatment initiated
2013	Chronic plantar fasciitis with marked functional limitation	No definitive diagnosis explaining the symptoms	Symptomatic management only; no improvement
2011–2017	Persistent tonsillar stones and intermittent throat symptoms	Repeated ENT consultations	Tonsillar findings considered benign; no targeted therapy
Early 2018	Acute worsening after neck massage; low-grade fever; ankle and wrist arthralgia; exertion-induced inflammatory pain	X-ray, cranial MRI, ankle MRI	No structural or inflammatory abnormalities identified; symptomatic treatment
Late 2018	Headaches on forward bending; palpitations and tachycardia	Cardiology evaluation	Normal findings; conservative management
2018–2019	Progressive musculoskeletal symptoms	Rheumatology evaluation	Diagnosed with seronegative polyarthritis; later undifferentiated autoimmune syndrome
January 19	Persistent throat complaints	Throat swab positive for Aspergillus fumigatus	Antifungal therapy; no clinical improvement
2019	Severe spinal pain; impaired ambulation; episodic dyspnea	Considered fibromyalgia, SLE, and ankylosing spondylitis	Immunosuppressive and antimalarial therapy was proposed but declined during breastfeeding
2019	Hair loss, nasal ulcers, electrolyte abnormalities	Laboratory evaluation	Magnesium and potassium supplementation; symptomatic relief of palpitations only
July 19	Identification of chronic tonsillitis as a focal infection	Targeted ENT reassessment	Tonsillectomy performed
Post-2019–2025	Gradual resolution of all systemic symptoms	Long-term follow-up	Complete and sustained recovery without recurrence

Summary of ENT examinations

Between 2009 and 2018, the patient attended 10 ENT consultations, and an additional eight during 2018-2019, averaging 1.8 visits per year. The primary reason was persistent tonsillar stones. Some ENT specialists removed them, while others ignored them or prescribed antibiotics, which proved ineffective. Several ENT physicians attributed her symptoms to reflux and recommended inhaled fusafungine (Bioparox), Cataflam, Ulcogan, alkaline gargling, chamomile tea, or lifestyle modifications. One of these physicians was the first to identify a tonsillar focal infection in 2011, when purulent material could be expressed from the tonsils, but by that time, she advised treatment for reflux.

Suspicion of Lyme disease

Repeated IgM-positive ELISA tests for Borrelia prompted the patient to visit the Center for Tick-borne Diseases in Budapest. Based on our laboratory results, we excluded both active and long-standing past Lyme disease. Drawing on our experience with hundreds of similar cases, we initiated a targeted ENT evaluation in Hungary and concluded that her decade-long symptoms originated from chronic tonsillitis. Ultimately, a tonsillectomy was performed in July 2019. Just before the operation, a rheumatologist evaluated the patient and agreed on the need for tonsillectomy but expressed doubt that it would improve systemic symptoms. Nevertheless, the patient experienced gradual and complete recovery and remains symptom-free to date (end of 2025).

Laboratory findings

Laboratory data were available for 2018-2021 but were incomplete. Most routine parameters, including serum iron, ferritin, C-reactive protein (CRP), thyroid-stimulating hormone (TSH), rheumatoid factor (RF), liver and kidney function tests, blood counts, immunoglobulins, hepatitis serologies, HLA-B27, and multiple autoantibodies, remained within normal limits.

Some notable abnormalities included a mildly elevated erythrocyte sedimentation rate (ESR) (on four occasions; 10 mm/h preoperatively; 7 mm/h one year postoperatively), low transferrin saturation, mild hypokalemia, high vitamin B12, antinucleolar antibody (ANA) initially positive (homogeneous, later granular), then negative by 2021 post-tonsillectomy. Leukocytosis occurred once but was otherwise normal. Lyme serology included inconsistent ELISA results (one IgM equivocal, two IgM-positive), while IgG was always negative. Overall, laboratory tests did not indicate sustained systemic inflammation, aside from minor and intermittent deviations. A detailed account of pathological laboratory results is shown in Table 2.

**Table 2:** Pathological laboratory results ESR: erythrocyte sedimentation rate; ANA: antinucleolar antibody; WBC: white blood cell count

Laboratory test	Result	Reference range
ESR	28 mm/h	5-20 mm/h
Transferrin saturation	14.1%	20-45%
Serum potassium	3.28 mmol/l	3.5-5 mmol/l
ANA	1:80-1:160	Negative
Vitamin B12	1018 pg/ml	197-771 pg/ml
WBC	10.8 G/L	4.0-10.0 G/L

## Discussion

This report emphasizes the clinical concepts outlined in our previously published article [11]. The main point is that there is no consensus on the diagnosis and management of chronic tonsillitis, either in routine clinical practice or in published guidelines, which are often contradictory and lack strong evidence [2]. The literature commonly distinguishes pus, detritus, keratin cysts, and tonsilloliths, implying that each requires different management. Except for pus, the other forms are generally considered benign [1]. The findings in this case suggest that these manifestations may be different expressions of the same biological process, each containing a significant bacterial load that could contribute to systemic symptoms, even when local inflammatory signs are absent. These observations highlight the importance of distinguishing relapsing acute bacterial tonsillitis from chronic tonsillitis to enhance diagnostic accuracy and efficiency (see Appendices). Guidelines should be revised to clearly reflect these distinctions if further research confirms these findings.

Several physicians linked the sore throat to gastroesophageal reflux and recommended treatments including Bioparox spray, Cataflam, Ulcogan, gargling with baking soda and chamomile tea, and following a reflux diet. Diagnostic uncertainty is highlighted by the identification of a tonsillar focal infection in 2011, when a physician noted purulent material from the tonsils, even though the same otolaryngologist, years later, continued to view reflux as the primary cause. This case demonstrates that failing to perform manual tonsillar compression can allow chronic tonsillitis to go undetected for years, resulting in unnecessary diagnostic procedures, misattribution of symptoms, and prolonged morbidity, despite the presence of an infection that could be cured surgically. Clinicians should clearly explain the serious risks of focal infections to patients to encourage consideration of tonsillectomy.

Observations from a specialized outpatient service dedicated to tick-borne diseases, with several decades of clinical experience, indicate that many patients referred with similar symptoms actually may have focal infections that are detectable by tonsillar compression and dental examination. It is frequently observed that such patterns are not recognized by clinicians and that patients may be referred for Lyme disease testing due to nonspecific systemic symptoms. An increase in testing may elevate the likelihood of false-positive results, reinforcing misconceptions regarding Lyme disease. Nevertheless, Lyme disease presents characteristic clinical features that can generally be distinguished from the systemic manifestations of focal infections [12].

Although no causal associations can be derived from this single case study, the fact that the patient’s symptoms worsened dramatically after a neck massage, possibly dislodging microbes from the tonsils into the bloodstream, and that her symptoms improved rapidly after tonsillectomy, may support a causal relationship. Nevertheless, further studies are required to determine whether this association is confirmed.

## Conclusions

Chronic tonsillitis, tonsillar stones, tonsillar debris, and detritus represent forms of focal infection capable of causing systemic complications. Chronic tonsillitis differs fundamentally from recurrent acute tonsillitis: while the latter responds to antibiotic treatment, the chronic form requires surgical removal of the tonsils. Failure to distinguish between these two entities can have serious consequences. This report highlights the underrecognition of oral focal infections in clinical practice and raises important questions about why oral abscesses can remain asymptomatic for years and what triggers their acute systemic manifestations. Given the large number of similar cases observed at the Center for Tick-borne Diseases, these questions warrant further investigation through systematic clinical and microbiological studies with more robust methodological support than single-case analyses.

## Appendices

**Table 3:** Comparison between recurrent acute bacterial and chronic tonsillitis This table highlights the fundamental clinical differences between recurrent acute bacterial and chronic tonsillitis, demonstrating that they are distinct conditions and should not be conflated in diagnostic or surgical decision-making [1,3,11]

Feature	Acute bacterial tonsillitis	Chronic tonsillitis
Throat pain	Severe throat pain	No sore throat unless a large tonsillolith causes pressure
Fever	High fever	No fever or low-grade fever only
Tonsillar appearance	Markedly inflamed tonsils	No erythema or acute swelling
Exudate	Abundant purulent exudate	Purulent material detectable only by tonsillar compression
Laboratory markers	Elevated inflammatory markers	No laboratory abnormalities
Response to antibiotics	Responds to antibiotics	Never or temporarily responds to antibiotics
Curative treatment	Antibiotic	Tonsillectomy

## References

[1] Nitek S, Legatowicz-Koprowska M, Szymański K, Chmielik LP (2017). Differential diagnosis of tonsillitis, tonsillar detritus accumulation, and tonsillar keratin cysts. Med Res Arch.

[2] Mesolella M, Cimmino M, Di Martino M, Criscuoli G, Albanese L, Galli V (2004). Tonsillolith. Case report and review of the literature. Acta Otorhinolaryngol Ital.

[3] Smith KL, Hughes R, Myrex P (2023). Tonsillitis and tonsilloliths: diagnosis and management. Am Fam Physician.

[4] Stoodley P, Debeer D, Longwell M, Nistico L, Hall-Stoodley L, Wenig B, Krespi YP (2009). Tonsillolith: not just a stone but a living biofilm. Otolaryngol Head Neck Surg.

[5] Adeniji-Sofoluwe AT, Obajimi MO, Oluwasola AO, Soyemi TO (2013). Mammographic parasitic calcifications in South West Nigeria: prospective and descriptive study. Pan Afr Med J.

[6] Póvoa A, Vieira P, Silva A, Pantazi I, Correia J (2021). Disseminated cysticercosis. Eur J Case Rep Intern Med.

[7] Nash TE, Bustos JA, Garcia HH (2017). Disease centered around calcified Taenia solium granuloma. Trends Parasitol.

[8] Delgado BJ, Bajaj T (2025). Ghon Complex. https://www.ncbi.nlm.nih.gov/books/NBK551706/.

[9] Tunkel AR, Korman MG (2013). Radiology of CNS infections. Insights Imaging.

[10] Guntinas-Lichius O, Geißler K, Mäkitie AA (2023). Treatment of recurrent acute tonsillitis-a systematic review and clinical practice recommendations. Front Surg.

[11] Lakos A, Nagy G, Varga Z, Rásonyi-Kovács P, Hornok S (2025). Reassessing chronic Lyme disease and post-treatment Lyme diseases syndrome as focal infections. Cureus.

[12] Lantos PM, Rumbaugh J, Bockenstedt LK (2021). Clinical practice guidelines by the Infectious Diseases Society of America (IDSA), American Academy of Neurology (AAN), and American College of Rheumatology (ACR): 2020 guidelines for the prevention, diagnosis and treatment of Lyme disease. Clin Infect Dis.

